# Factors associated with the increased incidence of necrotising enterocolitis in extremely preterm infants in Sweden between two population-based national cohorts (2004–2007 vs 2014–2016)

**DOI:** 10.1136/archdischild-2023-325784

**Published:** 2023-10-03

**Authors:** Pontus Challis, Karin Källén, Lars Björklund, Anders Elfvin, Aijaz Farooqi, Stellan Håkansson, David Ley, Mikael Norman, Erik Normann, Fredrik Serenius, Karin Sävman, Lena Hellström-Westas, Petra Um-Bergström, Ulrika Ådén, Thomas Abrahamsson, Magnus Domellöf

**Affiliations:** 1 Department of Clinical Sciences, Pediatrics, Umeå University, Umeå, Sweden; 2 Department of Clinical Sciences, Obstetrics and Gynecology, Lund University Faculty of Medicine, Lund, Sweden; 3 Department of Clinical Sciences, Lund, Paediatrics, Lund University, Lund, Sweden; 4 Department of Pediatrics, Institute of Clinical Sciences, University of Gothenburg Sahlgrenska Academy, Gothenburg, Sweden; 5 Department of Pediatrics, Sahlgrenska University Hospital, Goteborg, Sweden; 6 Division of Pediatrics, Department of Clinical Science, Intervention, and Technology, Karolinska Institutet, Stockholm, Sweden; 7 Department of Neonatal Medicine, Karolinska University Hospital, Stockholm, Sweden; 8 Department of Women's and Children's Health, Uppsala University, Uppsala, Sweden; 9 Department of Pediatrics, Södersjukhuset, Karolinska Institutet, Stockholm, Sweden; 10 Clinical Science and Education at Södersjukhuset, Karolinska Institute, Stockholm, Sweden; 11 Departments of Biomedical and Clinical Sciences and Pediatrics, Linköping University, Linköping, Sweden; 12 Department of Pediatrics, Linköping University Hospital, Linköping, Sweden

**Keywords:** Neonatology, Gastroenterology

## Abstract

**Objective:**

To investigate potential risk factors behind the increased incidence of necrotising enterocolitis (NEC) in Swedish extremely preterm infants.

**Design:**

Registry data from two population-based national cohorts were studied. NEC diagnoses (Bell stage ≥II) were validated against hospital records.

**Patients:**

All liveborn infants <27 weeks of gestation 2004–2007 (n=704) and 2014–2016 (n=895) in Sweden.

**Main outcome measures:**

NEC incidence.

**Results:**

The validation process resulted in a 28% reduction of NEC cases but still confirmed a higher NEC incidence in the later epoch compared with the earlier (73/895 (8.2%) vs 27/704 (3.8%), p=0.001), while the composite of NEC or death was lower (244/895 (27.3%) vs 229/704 (32.5%), p=0.022). In a multivariable Cox regression model, censored for mortality, there was no significant difference in early NEC (0–7 days of life) between epochs (HR=0.9 (95% CI 0.5 to 1.9), p=0.9), but being born in the later epoch remained an independent risk factor for late NEC (>7 days) (HR=2.7 (95% CI 1.5 to 5.0), p=0.001). In propensity score analysis, a significant epoch difference in NEC incidence (12% vs 2.8%, p<0.001) was observed only in the tertile of infants at highest risk of NEC, where the 28-day mortality was lower in the later epoch (35% vs 50%, p=0.001). More NEC cases were diagnosed with intramural gas in the later epoch (33/73 (45.2%) vs 6/26 (23.1%), p=0.047).

**Conclusions:**

The increase in NEC incidence between epochs was limited to cases occurring after 7 days of life and was partly explained by increased survival in the most extremely preterm infants. Misclassification of NEC is common.

**WHAT IS ALREADY KNOWN ON THIS TOPIC**

- Low gestational age is the primary risk factor for necrotising enterocolitis (NEC).
- A recent Swedish national cohort study of extremely preterm infants showed an increased NEC incidence over 10 years.
- During the same period, mortality and the incidence of other major neonatal morbidities decreased.

**WHAT THIS STUDY ADDS**

- Survival of the most premature infants seems to be the major driving factor behind the increase in NEC incidence.
- Early and late NEC have different risk factors, and only late NEC has increased over time.
- The misclassification of NEC in healthcare records and registers remains a concern.

**HOW THIS STUDY MIGHT AFFECT RESEARCH, PRACTICE OR POLICY**

- Future studies are needed to explore how risk factors and early and late NEC pathogenesis differ.
- Future studies are needed to address how to prevent NEC in the most preterm infants.
- Healthcare providers and registers have to improve the classification and validation of the NEC diagnosis.

## Introduction

Necrotising enterocolitis (NEC) is a devastating disease in preterm infants,^1 2^ with severe implications for the infant, family and healthcare system.^3 4^ The mortality is 20–30%, and the outcome is worse when surgery is required.^5–7^ Survivors have an increased risk for neurodevelopmental impairment.^8 9^ Low gestational age (GA) is the primary risk factor for NEC.^7 10^ The NEC incidence usually remains invariable over time in populations with an unchanged mean GA.^6^

The NEC incidence in Sweden tripled between 1987 and 2009, likely attributed to the increased survival of extremely preterm (EPT) infants.^11^ Over the last two decades, two national population-based 3-year cohort studies (2004–2007 and 2014–2016) included infants born before 27 weeks of gestation.^12 13^ The 1-year survival was significantly higher in the latter cohort, while the incidence of other neonatal morbidities was unchanged or decreased, except for NEC, which increased significantly among 1-year survivors from 6% to 10%.^13^

The aim of this study was to identify factors explaining the increased incidence of NEC in EPT infants in Sweden between 2004–2007 and 2014–2016. Our primary hypothesis was that improved survival in the most preterm infants would drive the rise in NEC incidence. Furthermore, we hypothesised that there might be an overestimation of NEC incidence due to misclassification in register data. Therefore, we decided first to perform a validation of the NEC diagnosis.

## Methods

### Participants

Epoch 1, the Extremely Preterm Infants in Sweden Study (EXPRESS) cohort (infants born April 2004 to March 2007), included 704 liveborn infants, of which 26 had a validated NEC diagnosis.^14^ Epoch 2, the EXPRESS 2 cohort (January 2014 to December 2016), included 895 liveborn infants. The two cohorts have previously been described in detail.^12 13^ During epoch 1, neonatal and perinatal data were prospectively collected by local investigators. During epoch 2, the Swedish Neonatal Quality Register^15^ was used for the primary data collection. In a second step, mortality and major morbidity data were cross-checked against medical records.^13^

### NEC diagnosis validation

NEC was defined as Bell stage II or higher in both epochs.^16^ Clinical data, including macroscopic and biopsy results from surgery and autopsy, were used to confirm NEC diagnosis. Spontaneous intestinal perforation (SIP) was recorded as a separate entity.

NEC cases in epoch 1 were previously validated against hospital records.^14^ At that time, nine cases of suspected NEC were reclassified because they did not fulfil the X-ray criteria. These nine cases were revalidated in the present study to achieve a uniform NEC classification in the two cohorts, resulting in one of these cases being reclassified from no NEC to NEC. For epoch 2, a similar validation was performed using a predefined standardised protocol (online supplemental table S1). For each reported NEC case, medical records were scrutinised. Diagnostic criteria for NEC included: typical findings of NEC at surgery or autopsy, NEC confirmed by biopsy, suggestive abdominal symptoms together with intramural or portal gas on abdominal X-ray or ultrasound. In addition NEC treatment and diagnostic characteristics were collected. Four undiagnosed NEC cases were added to the original epoch 1 study after examining the healthcare records of infants with significantly reduced enteral feeds.^14^ This was not feasible for epoch 2 due to a paucity of feeding data.

10.1136/fetalneonatal-2023-325784.supp1 Supplementary data

The NEC cases for epoch 1 were independently validated by a senior neonatologist and a resident in neonatology.^14^ In contrast, a senior neonatologist and coauthor independently validated the NEC cases for epoch 2 at each of the six study centres.

Background characteristics, mortality and morbidity have been previously described.^13^ Birth weight *z*-score was calculated from an intrauterine growth chart.^17^ Sepsis was confirmed by positive blood culture. Patent ductus arteriosus was diagnosed if requiring medical or surgical treatment. Retinopathy of prematurity (ROP) stages 3–5 was defined according to International Classification of Retinopathy of Prematurity-2,^18^ intraventricular haemorrhage (IVH) grades 3–4 according to Papile *et al* 19 and severe bronchopulmonary dysplasia (BPD) as treatment with ≥30% oxygen at 36 weeks’ postmenstrual age.^20^

### Missing data

Both data sets have previously been manually completed for missing data using hospital records.^12 13^ Data completeness was high, 98% for epoch 1 and 95% to 99% for epoch 2.^15^ Due to the small amount of missing data, listwise deletion was used and no imputation was performed.

### Statistics

Welch’s unequal variance t-test was used to compare means, and the Wilcoxon rank-sum test was used for medians. The Pearson’s χ^2^ test was used to compare categorical variables. The log-rank test was used for group comparison in the survival model. Cox regression was used to analyse the effects of risk factors on NEC while considering variable observation periods due to early mortality.

Propensity score stratification was used to determine if the increased NEC incidence was confined to infants with the highest risk of NEC. The score was created using a Cox regression model with NEC as the dependent variable. Perinatal risk factors for NEC were used as covariates for the Cox model: GA, birth weight z-score (SD), Apgar score at 5 min, caesarean delivery, prenatal corticosteroid treatment and multiple pregnancy. The propensity score was used to classify newborns into three risk groups and the rate of NEC occurrence was compared between epochs for each risk tertile.

Statistical analyses were conducted in R V.4.2.1 (R Project for Statistical Computing).

## Results

A total of 704 infants were included in epoch 1 and 895 in epoch 2, all liveborn at less than 27 weeks of gestation (table 1).

**Table 1:** Background characteristics, mortality and morbidity in extremely preterm infants in epoch 1 and epoch 2

	Epoch 1 (n=704)	Epoch 2 (n=895)	P value¶
Birth weight, g, mean (SD)	740.8 (183.9)*	718.1 (175.8)*	**0.013**
Birth weight z-score, mean (SD)	−0.8 (1.3)*	−0.9 (1.4)*	0.1
Gestational age, weeks, mean (SD)	25.0 (1.3)	24.9 (1.3)	0.080
Small for gestational age, n (%)	114 (16.3)*	166 (18.6)*	0.2
Male, n (%)	386 (54.8)	510 (57.0)	0.4
Apgar score at 5 min, median (IQR)	7 (5–9)	6 (4–8)†	**<0.001**
Caesarean delivery, n (%)	355 (50.4)	493 (55.1)*	0.06
Prenatal corticosteroid treatment, n (%)	588 (87.4)‡	785 (90.4)‡	0.06
Multiple pregnancy, n (%)	158 (22.4)	214 (23.9)	0.5
Maternal age, years, mean (SD)	30.8 (6.0)*	31.0 (6.0)*	0.5
Mechanical ventilation, days, mean (SD)	12.9 (16.6)§	19.9 (27.1)§	**<0.001**
Severe BPD, n (%)**	117/508 (25.3)§	115/716 (16.2)*	**<0.001**
Intraventricular haemorrhage††	221/565 (39.5)*	306/805 (39.0)‡	0.9
Intraventricular haemorrhage grades 3–4††, n (%)	40/565 (7.2)*	59/805 (7.5)‡	0.8
Retinopathy of prematurity stages 3–5‡‡, n (%)	177/514 (35.0)*	253/725 (35.9)‡	0.7
Treated patent ductus arteriosus, n (%)	343 (53.2)§	460 (51.4)	0.5
Sepsis, n (%)	269 (38.2)	322 (36.2)	0.4
Death within 1 year, n (%)	212 (30.1)	198 (22.1)	**<0.001**
Death within 24 hours of age, n (%)	104 (14.8)	49 (5.5)	**<0.001**
Death and/or NEC, n (%)	229 (32.5)	244 (27.3)	**0.022**
NEC Bell stages II–III, n (%)	27 (3.8)	73 (8.2)	**<0.001**
Early NEC (0–7 days), n (%)	14 (2.0)	21 (2.3)	0.6
Late NEC (>7 days), n (%)	12 (1.7)	52 (5.8)	**<0.001**
Macroscopic NEC§§, n (%)	21 (3.0)	57 (6.4)	**0.002**

Data are shown as mean (SD), median (IQR), or numbers and proportions (%). Bold denotes p<0.05.

*Missing n=1–10.

†Missing n=10–20.

‡Missing n=21–40.

§Missing n=41–60.

¶Welch’s two-sample t-test; Wilcoxon rank-sum test; Pearson’s χ^2^ test.

**Only including infants surviving PMA ≥36 weeks.

††Only including infants surviving ≥72 hours.

‡‡Only including infants surviving PMA ≥32 weeks.

§§Macroscopic NEC from autopsy or surgery.

BPD, bronchopulmonary dysplasia; NEC, necrotising enterocolitis; PMA, postmenstrual age.

The validation of epoch 2 NEC cases resulted in 29 cases of 102 being reclassified as no NEC (online supplemental figure S1). The reclassified cases included sepsis (n=7), SIP (n=6), volvulus (n=2), bowel atresia (n=1), meconium ileus (n=1), incarcerated inguinal hernia (n=1), circulatory disturbances (n=2), constipation (n=1) and Bell stage I or no NEC diagnosis (n=8).

**Figure 1:**
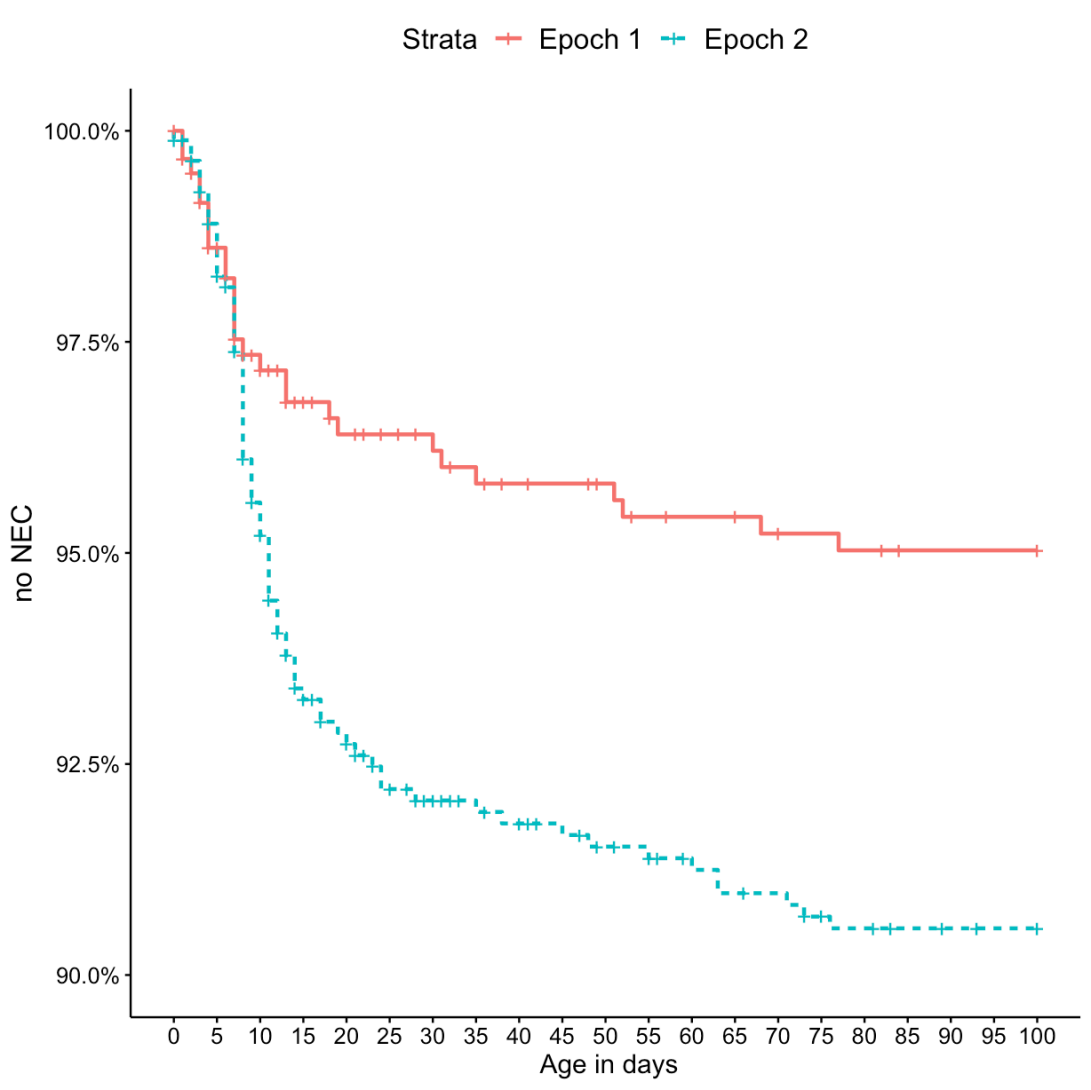
Kaplan-Meier curves displaying estimated probability for no necrotising enterocolitis (NEC) for both epochs. Each vertical tick mark in the curve indicates death. The curves are right censored at 100 days. Log-rank test was used for the p value.

The NEC incidence was higher in epoch 2 (8.2% vs 3.8%, p<0.001) (table 1, figure 1), but overall mortality was lower (1-year mortality 22.1% vs 30.1%, p<0.001) (table 1). The composite outcome of death within 1 year and/or NEC was lower in epoch 2 versus epoch 1 (27.3% vs 32.5%, p=0.022). Early NEC (0–7 days) did not differ between epochs (2.3% vs 2.0%, p=0.6), while the incidence of late NEC (>7 days) was higher in epoch 2 than in epoch 1 (5.8% vs 1.7%, <0.001) (table 1, figure 1). For each gestational week at birth, the NEC incidence was higher in epoch 2 than in epoch 1, while the non-NEC mortality was lower for each gestational week in epoch 2 (online supplemental figure S2). While the NEC incidence increased with lower GA at birth in epoch 2, instead in epoch 1, a lower NEC incidence was observed in gestational weeks 24 and below (online supplemental figure S3, panel A). The postmenstrual age at NEC occurrence was similar between epochs (median 185 days, IQR (179–206 days) vs median 184 days, IQR (175–198 days), p=0.2) (online supplemental figure S3, panel B). Apgar score at 5 min was lower in epoch 2 (median 6, IQR (4–8) vs median 7, IQR (5–9), p<0.001), while prenatal and maternal characteristics, birth weight *z*-score and neonatal morbidities except for NEC, mechanical ventilation in days and severe BPD did not differ significantly between epochs (table 1).

Significant risk factors from the univariate univariable Cox regression analyses (online supplemental table S2) were selected for a univariate multivariable model (table 2). The risk for NEC was not proportional over time, so Cox regression could not be used for the entire period. There was a steep risk increase in NEC in epoch 2 in the survival analysis (figure 1), suggesting the possibility of different risk factors, hence the division into early NEC (0–7 days) and late NEC (>7 days). In the multivariable model, there was no significant difference in early NEC between epochs (HR=0.9 (95% CI 0.5 to 1.9), p=0.9) (table 2), while the risk of late NEC was higher in epoch 2 (HR=2.7 (95% CI 1.5 to 5.0), p=0.001) (table 2). In addition to epoch, IVH remained an independent risk factor for late NEC. A low Apgar score at 5 min was the only significant risk factor for early NEC (table 2).

**Table 2:** Univariate multivariable HRs for early and late NEC

Risk factor	Early NEC (≤7 days) HR (95% CI)	Late NEC (>7 days) HR (95% CI)
	n=1421	n=1304
Epoch 2	0.9 (0.5–1.9), p=0.9	**2.7 (1.5–5.0), p=0.001**
Gestational age, weeks	0.9 (0.7–1.2), p=0.3	0.9 (0.7–1.1), p=0.3
Apgar score at 5 min	**0.85 (0.74–0.98), p=0.025**	NS
Caesarean delivery	0.6 (0.3–1.2), p=0.2	NS
Intraventricular haemorrhage	NS	**2.0 (1.2–3.2), p=0.008**

Cox regression for each outcome, censored for death for the composite of epoch 1 and epoch 2. Bold denotes p<0.05. Only significant covariates from the univariate univariable model are included (online supplemental table S1).

NEC, necrotising enterocolitis; NS, not significant in univariate univariable model.

Among NEC cases, GA and Apgar score at 5 min were lower in epoch 2 versus epoch 1 (online supplemental table S3), while birth weight, rate of small for gestational age or other neonatal outcomes did not differ (online supplemental table S3). The proportion of NEC infants diagnosed with intramural gas on X-ray was higher in epoch 2 (45.2% vs 23.1%, p=0.047), but there was no difference in macroscopic NEC (table 3).

**Table 3:** Clinical characteristics of NEC cases

	Epoch 1 (n=27)	Epoch 2 (n=73)	P value‡
Symptoms onset, age in days	7 (4–27)*	10 (7–17)	0.3
Symptoms onset stratified, age in days			0.4
Missing	1 (3.7)	0 (0)	
0–5 days, n (%)	8 (30.8)	14 (19.2)	
6–10 days, n (%)	7 (26.9)	24 (32.9)	
11–15 days, n (%)	2 (7.7)	15 (20.5)	
16–30 days, n (%)	3 (11.5)	9 (12.3)	
31 days or more, n (%)	6 (23.1)	11 (15.1)	
Early NEC (≤7 days)	14 (53.8)*	21 (28.8)	**0.022**
NEC onset at university hospital, n (%)	25 (96.2)*	58 (80.6)*	0.06
Intramural gas, n (%)	6 (23.1)*	33 (45.2)	**0.047**
Portal gas, n (%)	1 (4.0)*	12 (16.4)	0.1
Abdominal free gas on X-ray, n (%)	14 (53.8)*	29 (39.7)	0.2
Positive finding on X-ray or ultrasound, n (%)	17 (68.0)*	54 (74.0)	0.6
Surgical NEC¶, n (%)	14 (56)†	47 (64)	0.5
Time from symptoms to laparotomy¶, days	1 (1–2)†	1 (0–2)	0.4
Macroscopic NEC§, n (%)	21 (80.8)*	57 (78.1)	0.8
Confirming biopsy, n (%)			**0.010**
Missing	1 (3.7)	0 (0)	
Not obtained	14 (53.8)	24 (32.9)	
NEC confirmed	11 (42.3)	49 (67.1)	
NEC not confirmed	1 (3.8)	0 (0.0)	
Macroscopic NEC without positive X-ray or ultrasound, n (%)	8 (30.8)	15 (20.5)	0.3
Intestinal ostomy, n (%)	16 (64.0)*	41 (56.2)	0.5
Death within 1 year, n (%)	10 (37.0)	27 (37.0)	>0.9

Data are shown as median (IQR) or numbers and proportions (%). Bold denotes p<0.05.

*Missing n=1.

†Missing n=2.

‡Wilcoxon rank-sum test; Pearson’s χ^2^ test.

§Macroscopic NEC from autopsy or surgery.

¶Surgery within ≤3 days from NEC symptoms onset.

NEC, necrotising enterocolitis.

NEC cases had lower GA and had a higher incidence of IVH, ROP stages 3–5 and sepsis compared with infants without NEC (online supplemental table S4). Mortality was also higher, NEC versus no NEC (37.0% vs 24.9%, p=0.007) (online supplemental table S4).

In the propensity score analysis, the NEC incidence was significantly higher in epoch 2 versus epoch 1 only in the tertile of infants with the highest risk of NEC (34/291 (12%) vs 6/217 (2.8%), p<0.001), while there were no significant differences in the medium and low-risk tertiles (table 4). In the tertile of high-risk infants, who had an average GA of 23.6 weeks, the 28-day mortality was significantly lower in epoch 2 (35% vs 50%, p<0.001) (table 4).

**Table 4:** Risk factors, NEC and mortality among tertiles of infants based on NEC risk propensity score

Characteristic	Low			Medium			High		
	Epoch 1, n=228	Epoch 2, n=280	P value*	Epoch 1, n=227	Epoch 2, n=281	P value*	Epoch 1, n=217	Epoch 2, n=291	P value*
Gestational age, weeks	26.13	26.18	0.3	25.22	25.13	0.2	23.66	23.50	**0.044**
Birth weight z-score (SD)	−1.44	−1.60	0.2	−0.55	−0.74	0.06	−0.36	−0.31	0.7
Male, n (%)	101 (44)	120 (43)	0.7	135 (59)	177 (63)	0.4	133 (61)	189 (65)	0.4
Apgar score at 5 min	7.85	6.87	**<0.001**	6.89	6.22	**<0.001**	4.61	4.56	0.8
Caesarean delivery, n (%)	208 (91)	250 (89)	0.5	117 (52)	173 (62)	**0.023**	15 (6.9)	45 (15)	**0.003**
Prenatal corticosteroid treatment, n (%)	215 (94)	271 (97)	0.2	207 (91)	258 (92)	0.8	165 (76)	246 (85)	**0.016**
Multiple pregnancy, n (%)	35 (15)	58 (21)	0.1	57 (25)	69 (25)	0.9	61 (28)	73 (25)	0.4
Death within 28 days, n (%)	27 (12)	16 (5.7)	**0.014**	37 (16)	28 (10.0)	**0.034**	109 (50)	103 (35)	**<0.001**
Necrotising enterocolitis Bell stages II–III, n (%)	6 (2.6)	15 (5.4)	0.1	13 (5.7)	23 (8.2)	0.3	6 (2.8)	34 (12)	**<0.001**
Propensity score	−0.528	−0.514	0.3	−0.102	−0.100	0.9	0.459	0.472	0.6

Bold denotes p<0.05. Data are shown as mean or numbers and proportions (%).

The propensity score, which includes gestational age, birth weight z-score (SD), Apgar score at 5 min, caesarean delivery, prenatal corticosteroid treatment and multiple pregnancy, is divided into three risk tertiles: low, medium and high. Covariates, mortality and NEC incidence are shown for each epoch and tertile.

*Welch’s two-sample t-test; Pearson’s χ^2^ test.

NEC, necrotising enterocolitis.

## Discussion

The present study explored possible factors underlying the increase in NEC incidence in Sweden between 2004–2007 and 2014–2016. Even though the Cox regression model, censored for mortality, showed a remaining epoch difference in the incidence of late NEC, the propensity score analysis showed that the increase in NEC was limited to the high-risk group with the lowest GAs. Furthermore, the mortality in the high-risk group was significantly lower in epoch 2, and the overall risk of the composite outcome NEC or death was lower in epoch 2 in the whole study population. This suggests that increased survival of the most immature infants was an important contributing factor to the increased incidence of NEC. This is supported by the lack of an expected increase in NEC incidence in the lowest gestational weeks in epoch 1 (online supplemental figure S3, panel A).

The NEC incidence among infants <27 weeks was 8.2% in epoch 2, which was more than twice as high as in epoch 1. However, this is still in the range of other cohorts of EPT infants reporting incidences ranging between 5.4% and 13%.^21–24^

Increasing survival of preterm infants has been shown to increase the incidence of NEC.^1^ A Dutch cohort study described an increase in NEC incidence from 6.4% to 16% after implementing a new guideline for active treatment management of EPT infants.^25^

In contrast, two recent US multicentre cohort studies of EPT infants found lower NEC rates over time, despite increased survival.^26 27^ Improved neonatal care and increased use of breast milk feeding instead of formula were proposed explanations.^26^

Birth in epoch 2 remained a significant risk factor for late NEC in the Cox regression model after adjustment for GA and IVH. However, the Cox regression did not address whether NEC incidence and mortality differed between epochs in the group of most vulnerable infants; therefore, a propensity score analysis was added. In the propensity score analysis, the increased NEC incidence in epoch 2 was restricted to the high-risk tertile, where the 28-day mortality was significantly lower, suggesting that early mortality in high-risk infants with the lowest GAs during epoch 1 may have contributed to the difference in NEC incidence. However, we cannot exclude that residual confounding could explain part of the increased NEC incidence.

In Sweden, preterm practices have changed gradually between 2004–2007 and 2014–2016. However, these changes did not theoretically increase the risk of NEC as the rate of antenatal steroid treatment was consistently high, infants were routinely fed mother’s or donor milk and formula was rarely given before 34 weeks’ postmenstrual age.^28 29^ Probiotics and human milk-based fortifiers were not used for NEC prevention in Sweden during 2004–2016.^30^

The increased risk of NEC observed between the epochs was restricted to those who developed NEC after the first 7 days of life. It has been shown that age at the onset of NEC increases in a non-linear way with decreasing GA at birth,^31 32^ suggesting that different survival ratios between GA groups could be a factor. This is supported by our finding that the composite outcome of NEC or death was lower in epoch 2.

In our study, the risk factors for early NEC (first 7 days) differed from those for late NEC (after 7 days). A low Apgar score at 5 min was the sole significant risk factor for early NEC in the Cox regression model, while it was not a significant risk factor for late NEC. Previous observational studies have associated poor neonatal transition and low Apgar scores with NEC and mortality in EPT infants.^33–36^ However, findings have been inconsistent for NEC, and causality has been difficult to prove.^33^ Since NEC arises via different pathogenic pathways,^37^ our observation of different risk factors in early and late NEC cases may be important.

Sepsis and severe IVH were significantly associated with NEC. Sepsis could not be assessed as a risk factor for NEC since our data set did not allow us to determine the temporal relationship between the two diagnoses. Sepsis and IVH have been previously described as risk factors for NEC.^6 34 38^

Diagnostic accuracy for NEC improved in epoch 2 versus epoch 1, which might contribute to the higher NEC incidence during epoch 2 across all GAs. Positive findings were more frequent among NEC infants for the diagnostic modalities X-ray, ultrasound and biopsy in epoch 2. Ultrasound was seldom or never used for NEC diagnosis during epoch 1 but was commonly used during epoch 2, which may have contributed to improved diagnostics and may have affected the difference in NEC between epochs. The laparotomy rate was high in both epochs compared with other cohorts of very low birthweight infants.^6 26^

Misclassified NEC cases, especially overdiagnosis, were common in both epochs. After Bell stage I, SIP ranked second and third for the most common reason for misclassification in epoch 1 and epoch 2, respectively. Misclassifying SIP as NEC in clinical databases is well described,^39 40^ especially among EPT infants.^39 41^

A strength of this study was that both cohorts were population based, and data were prospectively collected. Both cohorts were rigorously validated. As many as 45 out of 140 NEC cases in the databases were misclassified, an important finding consistent with previous reports.^39 40^ However, this did not explain the differences in NEC incidence in this study. Even though both cohorts were population based and included births during two 3-year periods, they were not adequately powered to assess all risk factors associated with NEC. A limitation of the study is that data on feeding were unavailable for epoch 2, which may have resulted in a slightly lower incidence of NEC in epoch 2.

## Conclusion

NEC has increased significantly during the observed decade in Sweden. The increased NEC incidence is partly caused by increased survival among infants with the lowest GAs, but improved diagnostics and other factors cannot be excluded. The increase in NEC incidence was restricted to infants with onset after 7 days of age, and different risk factors were observed for early (0–7 days) and late (>7 days) NEC.

## Data Availability

No data are available. Due to Swedish personal data laws and regulations, data sharing is not possible for this study. These regulations prohibit the sharing of identifiable data without appropriate consent or legal basis.
