# Supplementary material for: Factors associated with the increased incidence of necrotising enterocolitis in extremely preterm infants in Sweden between two population-based national cohorts (2004–2007 vs 2014–2016)
Source: Arch Dis Child Fetal Neonatal Ed. 2023 Oct 3;109(1):87–93. doi: 10.1136/archdischild-2023-325784 (PMC10804036; doi:10.1136/archdischild-2023-325784)
Supplement: Supplementary data [file fetalneonatal-2023-325784supp001.pdf]

Supplemental Content

Risk factors for the increased incidence of necrotising enterocolitis in extremely preterm infants in Sweden: A comparison between two population-based national cohorts (2004-2007 vs 2014-2016).

Authors: Pontus Challis, Karin Källén, Lars J. Björklund, Anders Elfvin, Aijaz Farooqi, Stellan Håkanssson, David Ley, Mikael Norman, Erik Normann, Fredrik Serenius, Karin Sävman, Lena Hellström Westas, Petra Um Bergström, Ulrika Ådén, Thomas Abrahamsson, Magnus Domellöf

Table S1. NEC validation pro forma checklist

Table S2. Univariate univariable hazard ratios for early and late NEC

Table S3. Background characteristics, mortality and morbidity in infants with NEC

Table S4. Perinatal factors and morbidities among infants with and without NEC

Figure S1. Flow chart of data collection

Figure S2. Distribution of mortality and NEC for each gestational week.

Figure S3. NEC incidence for gestational age and postmenstrual age.

Table S1. NEC validation pro forma checklist

Region:

ID	X-ray/Ultrasound Findings				Clinical data		From Surgery or Autopsy			
Child-ID	Intramural Gas (Yes/No)	Porta Gas (Yes/No)	Abdominal Free Gas (Yes/No)	Date diagnostic X-ray (year-month-day)	Antibiotics (days)	Intestinal rest (days)	Laparotomy (Yes/No)	Macroscopisc NEC (Yes/No)	NEC according to biopsy (Yes/No)	Intestinal stoma (Yes/No)

Assessment					
NEC (Yes/No)	Bells Stage (2-3)	Onset date (year-month-day)	SIP (Yes/No)	Meconium ileus (Yes/No)	If not NEC, what diagnosis?

Table S2. Univariate univariable hazard ratios for early and late NEC

Risk factor	HR (95% CI) for early NEC (≤7d)	HR (95% CI) for late NEC (>7d)
Cohort Epoch 2	1.1 (0.6-2.2, p=0.8)	**2.9 (1.6-5.3, p=0.001)**
Birth weight, 100 grams	0.88 (0.72-1.08, p=0.2)	0.86 (0.75-1.00, p=0.052)
Gestational age, weeks	**0.75 (0.57-0.97, p=0.029)**	**0.72 (0.59-0.87, p=0.001)**
Birth weight, z-score (SD)	1.15 (0.87-1.50, p=0.3)	1.14 (0.93-1.39, p=0.2)
Small for gestational age	1.0 (0.4-2.3, p=0.9)	0.6 (0.3-1.3, p=0.2)
Male	1.6 (0.8-3.2, p=0.2)	1.0 (0.6-1.6, p=1.0)
Apgar score at 5 minutes	**0.83 (0.73-0.95, p=0.006)**	1.01 (0.91-1.13, p=0.86)
Cesarean delivery	**0.50 (0.25-0.99, p=0.045)**	0.63 (0.38-1.02, p=0.059)
Multiple pregnancy	0.9 (0.4-2.0, p=0.8)	1.2 (0.7-2.1, p=0.5)
Prenatal corticosteroid treatment	0.6 (0.2-1.6, p=0.3)	1.3 (0.4-4.2, p=0.6)
Intraventricular hemorrhage	Not relevant	**2.2 (1.3-3.6, p=0.002)**

Cox regression for each outcome censored for death. Bold denotes p<0.05.

Abbreviations: NEC, necrotising enterocolitis; HR hazard ratio; CI confidence interval

Table S3. Background characteristics, mortality and morbidity in infants with NEC

	Epoch 1 (N=27)	Epoch 2 (N=73)	P value[1]
Birth weight, grams	712 (639-917)	673 (585-800)	0.1
Gestational age, weeks	25.43 (24.71-25.71)	24.57 (23.57-25.71)	**0.048**
Birth weight, z-score (SD)	-0.78 (-1.39-0.07)	-0.46 (-1.52, 0.27)	0.5
Small for gestational age, n(%)	4 (14.8%)	10 (13.7%)	0.9
Male, n(%)	16 (59.3)	43 (58.9)	>0.9
Apgar score at 5 minutes	8.00 (6.00-10.00)	6.00 (5.00-7.00)	**0.004**
Cesarean delivery, n(%)	13 (48.1)	31 (42.5)	0.6
Prenatal corticosteroid treatment, n(%)	23 (92.0)*	67 (93.1)*	0.9
Intraventricular hemorrhage	15 (57.7)*	39 (54.9)*	0.8
Intraventricular hemorrhage grade 3-4, n(%)	5 (18.5)	18 (24.7)	0.5
Retinopathy of prematurity grade 3-5[2], n(%)	11/21 (52.4)	27/47 (57.4)	0.7
Treated patent ductus arteriosus, n(%)	15 (55.6)	34 (46.6)	0.4
Death within 24h of age, n(%)	0 (0.0)	1 (1.4)	0.5
Death within 1 year, n(%)	10 (37.0)	27 (37.0)	>0.9
Sepsis, n(%)	17 (63.0)	40 (54.8)	0.5

Data are shown as median and interquartile range (IQR) or numbers and proportions (%). Bold denotes p<0.05. Abbreviations: NEC, necrotising enterocolitis

* missing n=2 in epoch 1 and n=1 in epoch 2.

[1]Wilcoxon rank sum test; Pearson's Chi-squared test

[2]Only including infants surviving PMA ≥32w

Table S4. Perinatal factors and morbidities among infants with and without NEC.

	no-NEC (N=1499)	NEC (N=100)	P value[1]
Birth weight, grams	729.0 (180.8)*	715.1 (161.6)	0.4
Gestational age, weeks	25.0 (1.3)	24.8 (1.3)	0.10
Birth weight, z-score (SD)	-0.9 (1.4)*	-0.7 (1.3)	0.2
Small for gestational age, n(%)	266 (17.8)*	14 (14.0)	0.3
Male, n(%)	837 (55.8)	59 (59.0)	0.5
Apgar score at 5 minutes	6 (5-8)**	6 (5-8)	0.3
Cesarean delivery, n(%)	804 (53.7)*	44 (44.0)	0.06
Prenatal corticosteroid treatment, n(%)	1283 (88.9)***	90 (92.8)*	0.2
Intraventricular hemorrhage[2]	473/1271 (38.0)***	54/99 (55.7)*	**<0.001**
Intraventricular hemorrhage grade 3-4[2], n(%)	83/1271 (6.7)***	16/99 (16.5)*	**<0.001**
Retinopathy of prematurity stage 3-5[3], n(%)	392/1168 (34.4)***	38/71 (54.3)*	**<0.001**
Patent ductus arteriosus, n(%)	754 (52.4)****	49 (49.0)	0.5
Death within 1 year, n(%)	373 (24.9)	37 (37.0)	**0.007**
Sepsis, n(%)	534 (35.8)*	57 (57.0)	**<0.001**

Bold denotes p<0.05. Data are shown as mean and standard deviation (SD) or numbers and proportions (%).

Abbreviations: NEC, necrotising enterocolitis

* missing n=1-10, ** missing n=10-20, *** missing n=21-40 **** missing n=41-60

[1]Welch Two Sample t-test; Wilcoxon rank sum test; Pearson's Chi-squared test

[2]Only including infants surviving ≥72h

[3]Only including infants surviving PMA ≥32w

Figure S1. Flow chart of data collection

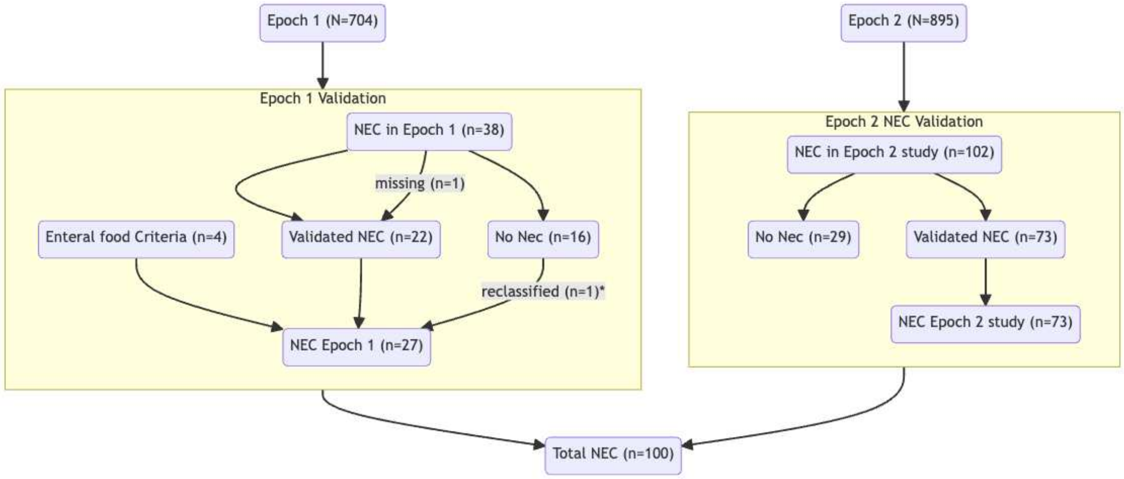

Study flow diagram of data collection and NEC diagnosis validation for both cohorts.

* Reclassified to achieve a uniform NEC classification in the two cohorts.

Abbreviations: NEC, necrotising enterocolitis

Figure S2. Distribution of Mortality and NEC for each gestational week.

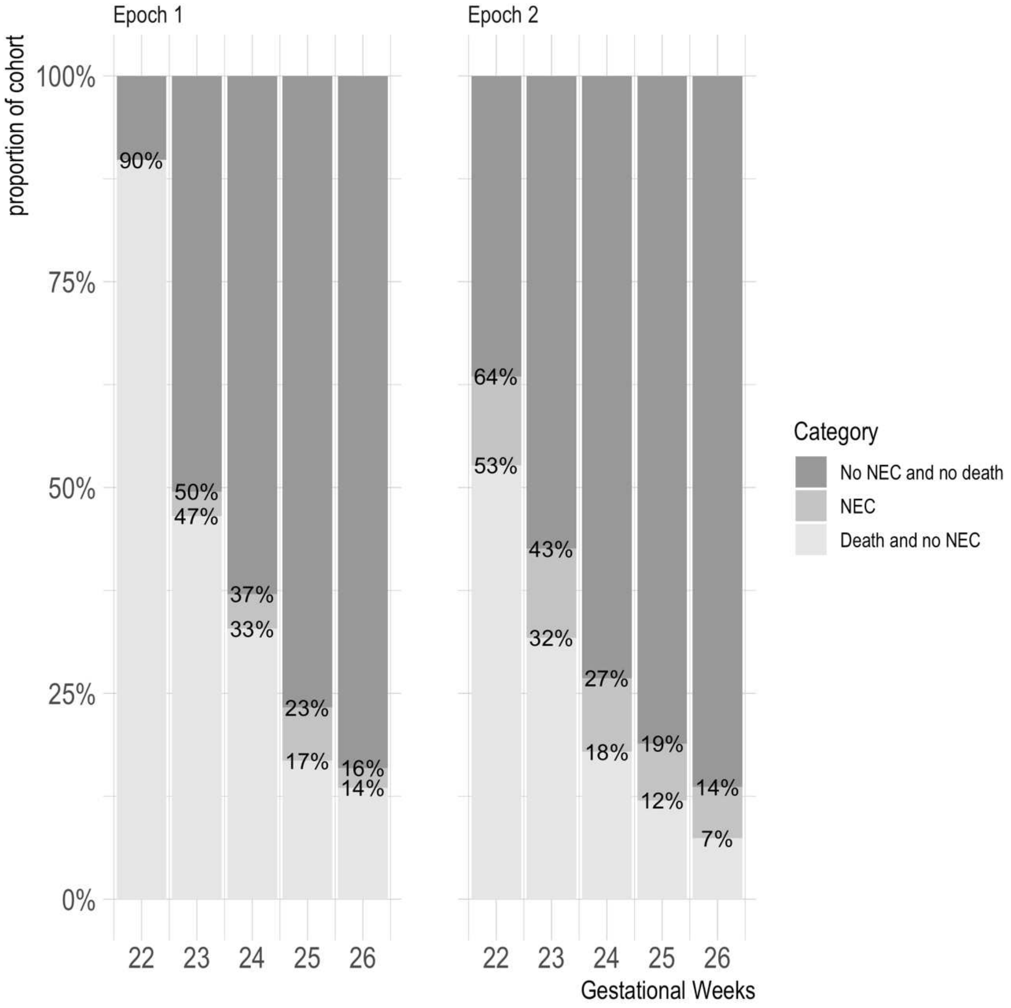

The proportion of death and no NEC, NEC and no NEC and no death for gestational weeks 22 to 26 for epoch 1 and epoch 2.

Abbreviations: NEC, necrotising enterocolitis

Figure S3. NEC incidence for gestational age and postmenstrual age.

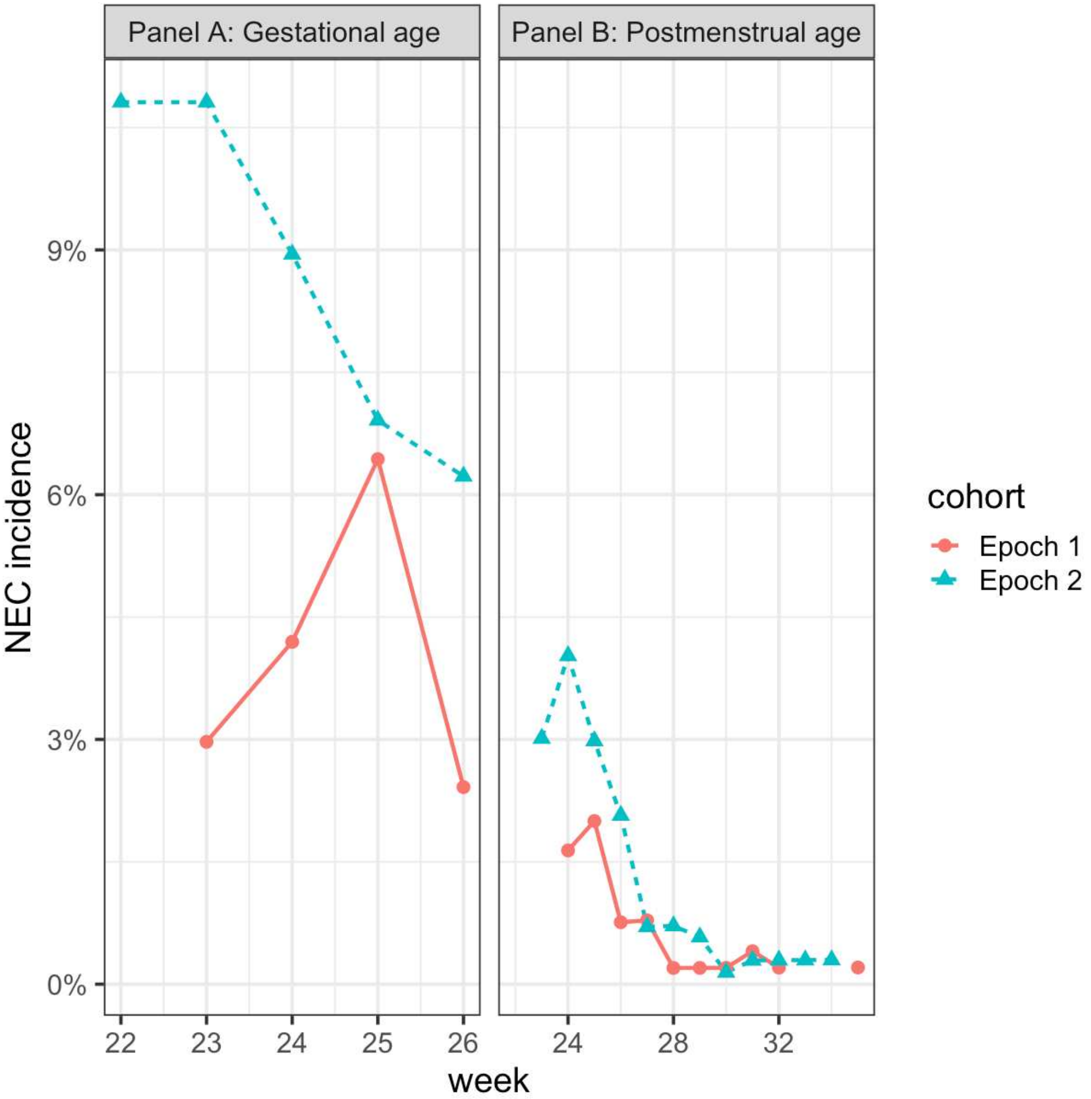

NEC incidence for gestational weeks 22 to 26 and post menstrual weeks 22 to 35 for epoch 1 and epoch 2.

Abbreviations: NEC, necrotising enterocolitis
